# Correlation between the binding affinity and the conformational entropy of nanobody SARS-CoV-2 spike protein complexes

**DOI:** 10.1073/pnas.2205412119

**Published:** 2022-07-15

**Authors:** Halina Mikolajek, Miriam Weckener, Z. Faidon Brotzakis, Jiandong Huo, Evmorfia V. Dalietou, Audrey Le Bas, Pietro Sormanni, Peter J. Harrison, Philip N. Ward, Steven Truong, Lucile Moynie, Daniel K. Clare, Maud Dumoux, Joshua Dormon, Chelsea Norman, Naveed Hussain, Vinod Vogirala, Raymond J. Owens, Michele Vendruscolo, James H. Naismith

**Affiliations:** ^a^Electron Bio-Imaging Centre, Diamond Light Source, Didcot OX11 0DE, United Kingdom;; ^b^Protein Production UK, The Research Complex at Harwell, Didcot OX11 OFA, United Kingdom;; ^c^Structural Biology, The Rosalind Franklin Institute, Didcot OX11 OQS, United Kingdom;; ^d^Yusuf Hamied Department of Chemistry, University of Cambridge, Cambridge CB2 1EW, United Kingdom;; ^e^Division of Structural Biology, University of Oxford, Oxford OX3 7BN, United Kingdom;; ^f^School of Biochemistry & Biotechnology, University of the Punjab, Lahore 54590, Pakistan

**Keywords:** COVID-19, electron microscopy, biophysics, affinity, computational tool

## Abstract

Camelid single-domain antibodies, also known as nanobodies, can be readily isolated from naïve libraries for specific targets but often bind too weakly to their targets to be immediately useful. Laboratory-based genetic engineering methods to enhance their affinity, termed maturation, can deliver useful reagents for different areas of biology and potentially medicine. Using the receptor binding domain (RBD) of the severe acute respiratory syndrome coronavirus 2 (SARS-CoV-2) spike protein and a naïve library, we generated closely related nanobodies with micromolar to nanomolar binding affinities. By analyzing the structure–activity relationship using X-ray crystallography, cryoelectron microscopy, and biophysical methods, we observed that higher conformational entropy losses in the formation of the spike protein–nanobody complex are associated with tighter binding. To investigate this, we generated structural ensembles of the different complexes from electron microscopy maps and correlated the conformational fluctuations with binding affinity. This insight guided the engineering of a nanobody with improved affinity for the spike protein.

## Significance

Understanding the structural principles that determine the binding affinity of nanobodies to the spike protein of severe acute respiratory syndrome coronavirus 2 has been difficult. We analyzed electron microscopy maps of nanobody-spike complexes and quantified the conformational entropy of binding. This informed the design of an engineered nanobody with improved binding to the spike protein. This result offers a guiding principle for the rational maturation of nanobodies directed against the spike. High-binding potency nanobodies have been shown to be effective in animal models; thus, this technology could have application in future pandemics.

Severe acute respiratory syndrome coronavirus 2 (SARS-CoV-2) is generally accepted to have originated from an animal reservoir, most likely bats, which after adaptive changes jumped the species barrier to infect humans in late 2019. Although the resulting coronavirus disease 2019 (COVID-19) is generally a mild respiratory disease in the young, it can be severe in elderly persons and those with comorbidities. The virus has spread around the globe; as of March 2022, it has resulted in nearly 18 million deaths (1), many more hospitalizations, and profound economic and social disruption. Vaccines have shown to be effective (2, 3). Repurposing of existing drugs, including the antiviral compound remdesivir and dexamethasone, has delivered benefits (4), with other drugs (molnupiravir, fluvoxamine, and Paxlovid) also showing significant promise, as reviewed by Wen et al. (5). Moreover, injection of monoclonal antibodies has shown promise in preventing serious disease (6). However, there remains strong interest in new therapies that reduce transmission and decrease disease severity, particularly ones that could be deployed rapidly. In addition, the virus has evolved to escape therapeutic monoclonal antibodies (7–9), so these therapeutics may need to be modified to remain active against emerging variants.

Isolating antibodies by naïve library screening is very rapid, requiring only the target antigen. Phage display methods (10) have been applied to diverse arrays of antibodies which by repeated cycling identify the strongest binders in an iterative process (11). However, binding strengths of the naïve library hits are usually not strong enough for the agents to be useful, which is perhaps unsurprising, as libraries can only sample a small portion of the available diversity. One solution to this problem is to improve the binding affinity through a process known as affinity maturation (12), where the potential recognition sites (usually the complementarity-determining regions [CDRs] of antibodies) of the initial binders are mutated and the strongest binding mutants are selected (13). Such laboratory-based approaches mimic the natural selection process that operates in mammals to produce high-affinity antibodies to foreign antigens (14).

The epitope binding site of single-domain antibodies derived from the heavy chain–only antibodies of camelids is contained within a single compact variable domain of about 130 amino acids (nanobody) (15, 16). Epitope recognition commonly uses CDR1 (typically residues 28 to 34), CDR2 (typically residues 48 to 54), and the longer CDR3 (typically residues 97 to 114). Nanobodies and their derivatives have a long history in structural biology, where they have contributed to many structural studies by stabilizing conformational states for both electron microscopy (EM) and crystallography (for a recent review, see ref. 17) or adding sufficient size to enable EM studies (18). There are already multiple studies of nanobodies that neutralize SARS-CoV-2 (19–23) as well as their use as tools for the structural study of viral proteins (24). Since nanobodies are inherently compact, they are particularly suitable for maturation approaches. As an alternative to maturation, very large (10^12^ sequences) synthetic libraries of binders, known as sybodies, have been screened to identify tight binders (25). Single-chain human antibodies have also been engineered and used against SARS-CoV-2 (26, 27). However, the application of computational approaches to maturation has been limited.

A recent review analyzed structures of human antibodies bound to their targets from HIV (28). Improved binding arose from the optimization of the complementarity between antibody and antigen, including through an increase in the surface area buried upon binding and in the rigidity of the loops preorganized for binding (28). Deep sequencing then allowed the pathway by which these mutations were selected to be reconstructed. However, there are very few systematic studies of nanobody selection and maturation with a single antigen, which is necessary if deep learning approaches are to be brought to bear. In this context, structural insight could be particularly helpful since it can identify those changes which directly and indirectly affect binding to the antigen.

To develop an understanding of the structural basis of nanobody binding affinity, we carried out the biophysical and structural characterization of a series of six nanobodies targeting the receptor binding domain (RBD) of SARS-CoV-2 spike protein, comprising a hit from screening a naïve nanobody library and five affinity-matured mutants derived from this parental binder; two of the mutants have been shown to be potently neutralize the virus (29). Using these data, we identified the key conformational properties that drive the affinity of this class of molecule. Based on cryo-EM data and a computational approach, we then engineered a hybrid nanobody with a CDR3 sequence that improved the binding affinity to the spike protein. We suggest that the quantitative estimate of the conformational entropy of the spike-nanobody complexes from experimental EM maps is helpful in the rational maturation of nanobodies against their target.

## Results

### The Nanobodies.

H11 was isolated from the naïve library and PCR shuffling led to the five matured agents which were purified (Table 1). Additional mutants were constructed and purified in the same way and are shown in Table 1.

**Table 1.** Amino acid sequences of the family of nanobodies described in this work

Nanobody	Amino acid sequence
H11	97 A QTRVTRS LLSDYATWPYDY 116
H11-A10	97 A **GFSA**TRS LLSDYATWPYDY 116
H11-H6	97 A **GSKI**TRS LLSDYATWPYDY 116
H11-B5	97 A **SYQA**TRS LLSDYATWPYDY 116
H11-D4	97 A **R**T**ENV**RS LLSDYATWPYDY 116
H11-H4	97 A QT**HYVSY** LLSDYATWPYDY 116
H11-H4 R52E	**R52E** 97 A QT**HYVSY** LLSDYATWPYDY 116
H11-H4 R52A	**R52A** 97 A QT**HYVSY** LLSDYATWPYDY 116
H11-H4 R52K	**R52K** 97 A QT**HYVSY** LLSDYATWPYDY 116
H11-H4 Y101A	97 A QT**HAVSY** LLSDYATWPYDY 116
H11-H4 Y104S	97 A QT**HYVS**S LLSDYATWPYDY 116
H11-H4 Y104F	97 A QT**HYVSF** LLSDYATWPYDY 116
H11-H4 T99Y (B5 hybrid)	97 A Q**YHYVSY** LLSDYATWPYDY 116
H11-H4 Q98R H100E (D4 hybrid)	97 A **R**T**EYVSY** LLSDYATWPYDY 116
H11-H4 Q98R T99Y H100E (B5 D4 hybrid)	97 A **RYEYVSF** LLSDYATWPYDY 116
H11 V101Y R103S	97 A QTR**Y**T**S**S LLSDYATWPYDY 116

Residues which differ from the H11 parent are shown in bold.

### Structural Biology of the Nanobody Complexes.

**Fig. 1.**
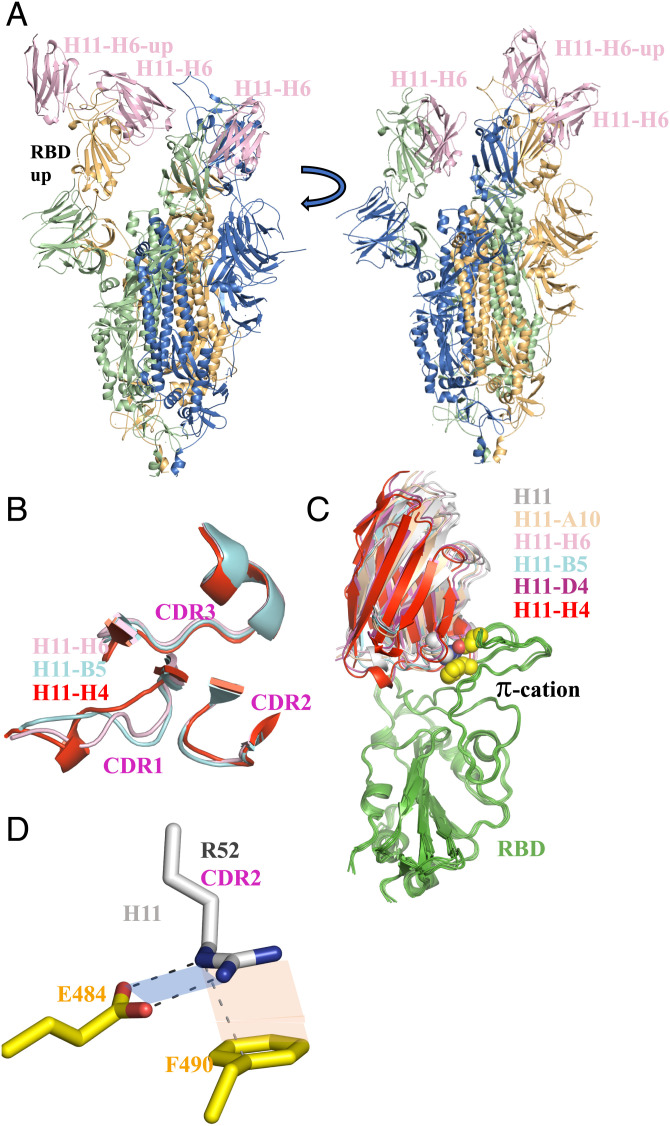
Cryo-EM structures of the nanobodies bound to the spike protein of SARS-CoV-2. (*A*) Cryo-EM structure of a nanobody (H11-H6) bound to spike. Nanobodies H11, H11-A10, and H11-B5 all give the same one-up–two-down conformation of the spike which was first described for nanobodies H11-H4 and H11-D4 (29). A second view (rotated 90° around the vertical axis) is shown. The sequence variation in the nanobodies is confined to a six-residue segment of the CDR3 loop (Table 1). (*B*) Although the residues that vary between the six nanobodies are located in the CDR3 region, structural changes are actually seen in CDR1. Shown are the three nanobodies which illustrate the different CDR1 structures observed. (*C*) Superposition of the RBD-nanobody complexes reveals the nanobodies bind in slightly different orientations on the surface of the RBD. The RBD molecule is colored green, and nanobodies are colored individually as follows: H11 (gray), H11-A10 (wheat), H11-H6 (light pink), H11-B5 (cyan), H11-D4 (purple), and H11-H4 (red). The complexes are anchored by the π–cation involving R52 from CDR2 of the nanobody (the side chain as spheres with carbon atoms colored white, nitrogen blue) interacting with E484 and F490 (carbon atoms yellow, oxygen atoms red) from RBD. (*D*) Close-up view of the π-cation interaction from the H11 complex. The geometry (distance and orientation) of both the salt bridge (blue plane) and the π stacking interaction (orange plane) varies the complexes (Table 2).

Single-sparticle cryo-EM confirmed that the parent H11 and variants H11-A10, H11-H6, and H11-B5 bound to the same one-up–two-down conformation of the spike that we observed for H11-H4 and H11-D4 (29) (Fig. 1*A*). As previously noted (29), this arrangement results in additional contacts between one nanobody bound to the down-configured RBD and a neighboring up-configured RBD. It was proposed that nanobody binding drives the spike into a single arrangement, removing the all-down conformer and various other ones (29). Since the resolution of the cryo-EM maps precluded a detailed analysis of the interactions, we carried out crystal structure determination of all nanobodies bound to RBD.

As might be expected from the small number of changes in sequence, the overall structures of the nanobodies are very similar. When judged by the number of residues that can be superimposed and by the root mean square deviation (RMSD) of these residues, H11, H11-D4, and H11-H4 are more similar to each other (RMSD of 0.4 to 0.6 Å over 126/127 Cα) than they are to the other three (RMSD of 0.5 to 0.9 Å over 122 to 126 Cα atoms). H11-A10 and H11-H6 are very similar to each other (0.4 Å over 127 Cα atoms), with H11-B5 being slightly different (0.6 to 0.7 Å over 125 Cα atoms) (*SI Appendix*, Table S3). Although the sequence changes are localized to CDR3, the structure of this region is essentially unchanged. However, these sequence changes in CDR3 do result in changes in the main chain structure of CDR1 (R27 to A33). H11, H11-D4, and H11-H4 adopt the same CDR1 conformation, while H11-A10, H11-H6, and H11-B5 have quite different CDR1 conformations (Fig. 1*B*). H11-A10 and H11-H6 are identical, but H11-B5 has a unique arrangement for R27 to F29. The change in CDR1 is a result of the mutation of Q98 in H11 to a smaller residue (G98 H11-A10, H11-H6, and S98 H11-B5), which creates a void. This void is filled by F29, which alters the structure of CDR1 but maintains the structure of CDR3 (Fig. 1*B* and *SI Appendix*, Fig. S2*A*). No such void is created in H11-D4 (R98) or H11-H4 (Q98) (30). Analysis of the B-factors shows that all CDRs are, on average, well ordered compared to the entire nanobody (*SI Appendix*, Table S4).

As expected, the nanobodies all recognize the same epitope on RBD; but when the complexes are superimposed (using the RBD to calculate the matrix), subtle changes in orientation are seen between the nanobodies (Fig. 1*C*). When the F2 nanobody was present (necessary to obtain crystals), this was bound remotely from the epitope of the H11 nanobodies. Analysis of the superposition of the RBD suggests that F2 has no systematic effect on the RBD structure in contact with the H11 nanobodies, consistent with previous observations (30). Recognition of the RBD by the nanobodies is dominated by CDR3 and CDR2, with CDR1 only making a few contacts (*SI Appendix*, Fig. S2*C*). Residues 100 to L106 (overlapping with the variable 99 to 104), D108, W112, and D115 of the CDR3 loop interact with RBD. In CDR2 residues, R52, S54, G55, S57, and A58 make contacts with the RBD. CDR1 of H11, H11-D4, and H11-H4 makes essentially no contact with RBD; while in H11-H6, H11-A10, and H11-B5, the residue T31 forms hydrogen bonds with R52 of the RBD (*SI Appendix*, Fig. S2*D*). A global comparison of the interfaces using PISA (31) and Interprosurf (32) shows that although H11-H4 buries the largest surface area, there is no discernible trend in the analysis that fits experimental binding data (*SI Appendix*, Table S6).

Since the global analysis of the interface was unable to rationalize affinity, we examined in detail the variable residues 99 to 104 of CDR3 for all six nanobodies that are in contact with RBD. The residue at 99 is found as an aromatic in H11-A10 (F99) and H11-B5 (Y99), and Y114 adopts a different position to avoid a clash (*SI Appendix*, Fig. S2*B*). In the other nanobodies, either a serine or threonine is found at this position where it makes an internal hydrogen bond to the backbone amide of residue 100 (*SI Appendix*, Fig. S2*B*). In H11, the side chain of R100 (H11-A10 S100, H11-H6 K100, H11-H4 H100) makes several contacts with RBD (*SI Appendix*, Fig. S2*C*). In H11-D4 and H11-B5, the side chain E100 (Q100 H11-B5) points away from RBD and makes an internal hydrogen bond. The side chain of residue 101 in all nanobodies makes similar van der Waals interactions with Y449 from RBD as described previously (30) (*SI Appendix*, Fig. S2*C*). Residue 102 is found either as valine (H11-H4, H11-D4) or threonine (Table 1), which forms van der Waals interactions with L452 of RBD similar to what has been described (30) (*SI Appendix*, Fig. S2*C*). T102 forms a hydrogen bond with S494 of RBD (*SI Appendix*, Fig. S2*C*). In all H11 nanobodies except H11-H4 (which has S103), R103 makes an internal salt bridge with D108, and an intramolecular π–cation interaction with W112 and S104 makes a hydrogen bond to the carbonyl of F490 that was described for H11-D4 (30). In H11-H4, S103 makes several internal hydrogen bonds but the side chain has no contact with RBD (30), while Y104 in H11-H4 forms a hydrophobic cluster with L455, F456, and Y489 of RBD (30).

In each structure, R52 in the invariant CDR2 makes a bidentate salt bridge with E484 and π–cation interaction with F490 of the RBD (Fig. 1*D*), as noted previously for H11-H4 (29). In H11-A10, H11-B5, and H11-H6, the carbonyl of T30 makes an additional hydrogen bond with the tip of R52 (*SI Appendix*, Fig. S2*D*). H11-D4 and H11-H4, which have a different arrangement of the CDR1 loop, cannot make this hydrogen bond and a water molecule instead satisfies the hydrogen bond (29). In H11, the water molecule appears to be missing, although this may be a feature of data resolution. The ideal distance for a bidentate salt bridge (measured between the O and N atoms) is around 2.6 Å with the carboxylate and guanidine coplanar (33). The ideal π–cation arrangement has the plane of the aromatic ring and the guanidine group parallel, with the center of the aromatic ring aligned with the central carbon of the guanidine ring with a separation of 3.3 Å (34). Analysis of the H11 structure revealed that the configuration of the π–cation salt bridge deviated from this ideal (Table 2). This prompted us to examine the interaction in all the structures (Table 2) and the data show a trend where H11 is least optimal, with H11-H4, closely followed by H11-D4, having the most optimal arrangement.

**Table 2.** Geometry of the π-cation salt bridge

Nanobody	R52 − E484			R52 − T31 (carbonyl)	R52 − F490	
	Distance (Å)		Dihedral (°)	Distance (Å)	Distance (Å)	π-Coplane (°)
	OE1 − NH2	OE2 − NE	NE-NH2-OE1-OE2	NH1 − O	NE – CG	
H11	2.7/2.9	2.9/2.9	19 /17	Missing	3.6/3.6	17/15
H11-A10	2.9/2.9	2.7/2.8	1/1	3.08/3.09	3.7/3.7	9/7
H11-B5	2.9/2.9	2.7/2.6	4/3	3.04/3.00	3.5/3.6	12/13
H11-H6	2.8	2.8	7	2.90	3.6	18
H11-D4	2.8	2.6	1	Water bridge	3.6	10
H11-H4	2.7	2.6	5	Water-water	3.6	5
H11-H4 Q98R H100E D4 hybrid	2.8	2.7	7	Water-water	3.5	6

### Biophysical Analysis of the Nanobodies.

**Fig. 2.**
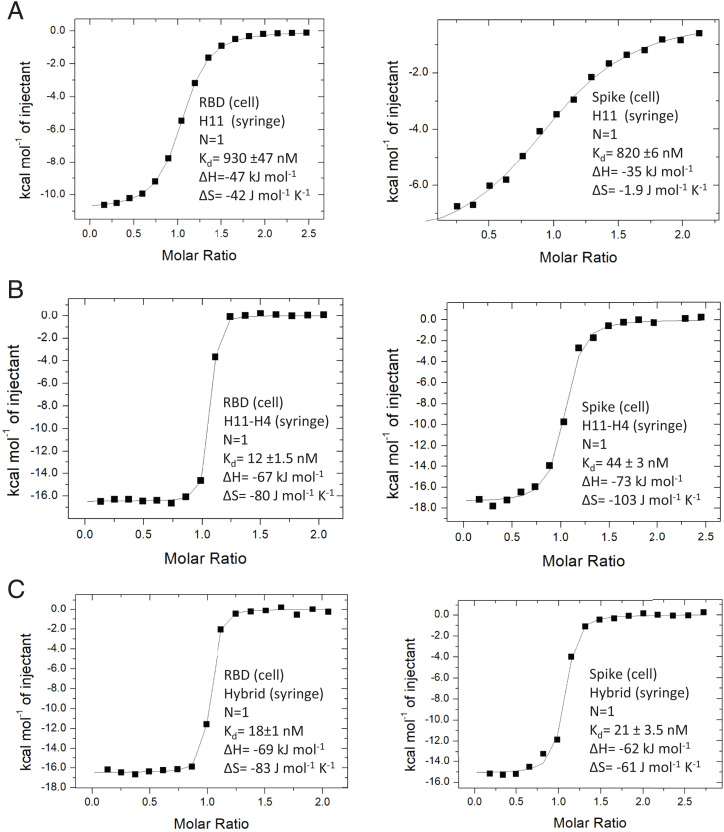
ITC analysis of binding. (*A*) The parent H11 nanobody binds weakly to both RBD (*Left*) and spike (*Right*). The binding constants were reported previously (30). (*B*) The tightest binding nanobody H11-H4 (ITC data previously reported) (29) binds more strongly to RBD (*Left*) than spike (*Right*). (*C*) The engineered D4 hybrid nanobody binds slightly more weakly than H11-H4 to RBD (*Left*), but binds more strongly to spike (*Right*). This is due to the smaller entropic penalty when engaging spike. Replicates are shown in *SI Appendix*, Fig. S4.

The nanobodies all have a denaturation temperature (T_M_) above 60 °C, with H11-H4 marginally less stable than the others (*SI Appendix*, Fig. S3 and Table S4). Using isothermal titration calorimetry (ITC; Fig. 2 and *SI Appendix*, Fig. S4) and surface plasmon resonance (SPR; *SI Appendix*, Fig. S5 and Table S5), we analyzed the interactions between RBD and the nanobodies (H11, H11-A10, H11-H6, H11-B5) and other mutated variants (*SI Appendix*, Fig. S5) as we employed for H11-H4 and H11-D4 (29). Despite the small number of changes in the nanobody sequences (Table 1), the range of binding affinities covers two orders of magnitude. In all cases, binding to RBD is enthalpically driven and entropically unfavorable. The “parent” nanobody, H11, binds the most weakly, having a much lower binding enthalpy than the other nanobodies. This is consistent with a poorly optimized π–cation interaction with the RBD (Table 2). The improvement in geometry of this interaction correlates with increased binding (Table 3). Site-directed mutation of R52 abolishes binding, confirming the central role of the π–cation interaction (*SI Appendix*, Fig. S6 *A*–*C*). By contrast, comparison between the thermodynamic properties of nanobody binding (Table 3) with the sequence changes in the CDR3 loop (Table 1) reveals that no single amino acid in the CDR3 variable six-residue cassette is critical for binding.

**Table 3.** Thermodynamic properties of nanobodies described in this work binding to the RBD

Nanobody	Thermodynamics (kJ/mol)				Kinetics
	K_D_ (nM)	ΔH	−TΔS	ΔG	t_1/2_ (s)
H11*	930 (±47)	−47	12	−34	NM
H11-A10	60 (±4.6)	−83	42	−41	14
H11-H6	57 (±4.5)	−80	39	−41	25
H11-B5	53 (±5.3)	−68	27	−41	14
H11-D4*	39 (±2)	−76	34	−42	26
H11-H4*	12 (±1.5)	−69	23	−46	29
H4_R52E	ND^†^	NP	NP	NP	NP
H4_R52E with E484R in RBD	ND^†^	NP	NP	NP	NP
H4_R52A	ND^†^	NP	NP	NP	NP
H4_R52K	ND^†^	NP	NP	NP	NP
H4_Y101A	1,079 ± 193	−42	8	−34	NP
H4_Y104S	409 ± 91	−46	9	−37	NP
H4_Y104F	40 ± 12	−57	15	−42	9
H11_V101Y_R103S	5,221	−46	16	−30	NP
H11-H4 T99Y (B5 single hybrid)^‡^	NM	NP	NP	NP	NP
H11-H4 Q98R H100E (D4 hybrid)	18 (±1)	−69	25	−44	29
H11-H4 Q98R, T99Y, H100E (B5-D4 hybrid)^‡^	NM	NP	NP	NP	NP

NM, not measured reliably; ND, no binding detected; NP, experiment not performed.

^*^These data are from previous publications (29).

^†^SPR did not detect any binding; no further analysis carried out.

^‡^SPR showed weak binding with KD > 1000 nM; no further analysis was carried out.

H11-A10 and H11-H6 behave very similarly, showing the large increase in binding enthalpy correlating with improvement in π–cation geometry. This gain is somewhat offset by an increase in the unfavorable entropy term compared to H11 (Table 3). This is a trend in the data consistent with the phenomenon of enthalpy-entropy compensation, where the structural changes in the binding molecules result in enthalpic and entropic changes that balance each other (35–37). Of note is that H11-H4, the strongest binder, has gained affinity by a lower entropic penalty.

H11-H6 has a notably slower off rate than H11-A10 and H11-B5 despite similar free energy of binding and the salt bridge π–cation arrangement (Table 3). In H11-H6, W112 adopts a nonfavored rotamer, due to its interaction with the side chain of I101. I101 is in turn held in position by an interaction with Y449 of RBD. We suggest that the slow off rate may reflect the reorganization of I101 and W112 (relative to the parent H11) that has occurred on binding. In H11-H4, the side chains of Y101 and W112 form π-stacking interactions which fix Y101 in a favored conformation, removal of this stacking interaction in the mutant Y101A reduces affinity by nearly 100-fold (Table 3 and *SI Appendix*, Fig. S4). We attempted to introduce this interaction into the parent H11 nanobody with the double mutant A101Y, R103S but failed to increase affinity (Table 3). In H11-H4, Y104 forms a hydrophobic cluster with RBD; the Y104F mutation, which would be predicted to preserve this cluster, does indeed largely retain affinity. The Y104S mutation, which would be predicted to disrupt the cluster, does reduce affinity (by around 30-fold).

### Computational Analysis of the Binding Affinity of the Nanobodies.

**Fig. 3.**
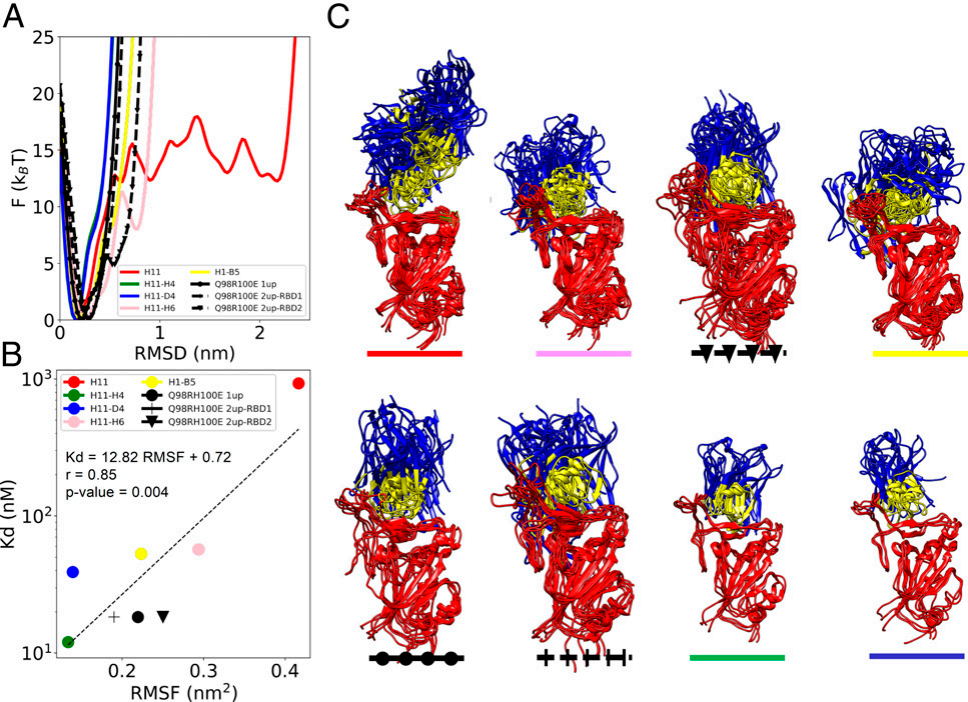
Correlation between the binding affinity and the conformational entropy of the nanobody-RBD complexes. (*A*) The dynamics of a nanobody-spike complex is represented as a free energy landscape as a function of the distance from the most populated state of the complex. (*B*) Correlation between the binding affinity (K_D_) and the conformational entropy of the complex, which is quantified through the root mean square fluctuations within the structural ensembles (RMSF). (*C*) Structural ensembles of the nanobodies in complex with the up RBD. The RBD is colored in red, the CDR regions in yellow, and the rest of the nanobody in blue.

To identify the structural determinants of the enthalpic and entropic contributions to the binding affinity of the nanobodies, we carried out a computational analysis of the ensembles of structures generated using the electron microscopy metainference (EMMI) method (38–40). These structural ensembles describe the conformational heterogeneity of the spike-nanobody complexes, as captured by the cryo-EM maps. This analysis showed that the parent nanobody H11 bound to the spike trimer exhibits a heterogenous ensemble of structures, indicated by multiple minima in its free energy landscape (Fig. 3 and *SI Appendix*, Fig. S1). This observation prompted us to investigate whether the nanobodies with higher binding affinity (H11-B5, H11-D4, H11-H4) could be associated with narrower free energy landscapes, which would correspond to a lower conformational entropy. As suspected, we found that the H11 variants reduced the dynamics of the nanobody-RBD complexes as the binding became stronger (Fig. 3 and *SI Appendix*, Fig. S1). As a measure of conformational heterogeneity, we used the root mean square fluctuation (RMSF) averaged over the three RBDs in the EM structure (Fig. 3). Our results indicate that the RMSF correlates positively with the K_d_, with a coefficient of correlation of 0.85 and a *P* value of 0.004. We also calculated the entropy using the Schlitter method (41) and see a good correlation (coefficient of correlation, 0.75; *P* value of 0.019) with our preferred approach. The reduction of dynamics for increasingly potent H11 variants is visualized in terms of structural ensembles in Fig. 3. Detailed analysis of the dynamics identified the residues in the CDR3 loop that influence the dynamics. This analysis led us to design three H11-H4 hybrids—***R***T***E***YVSY (D4 hybrid), ***RYE***YVSY (B5 D4 hybrid), and Q***Y***HYVSY (B5 hybrid)—by combining the sequence motifs corresponding to the lowest degree of conformational heterogeneity. We then used these hybrids as controls to support the hypothesis that binding affinity and conformational entropy are linked.

### Analysis of the Designed Nanobodies.

**Fig. 4.**
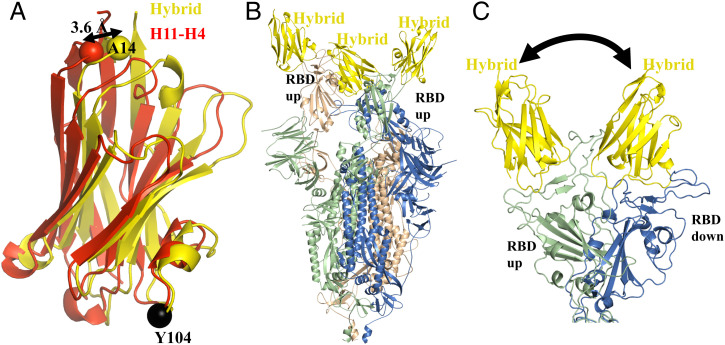
Cryo-EM structures of the engineered hybrid nanobody. (*A*) Superimposing the RBD complexes reveals that the hybrid nanobody (yellow) has pivoted at Y104 relative to H11-H4 (red). This results in a 3.6-Å shift at A14 which is distant from the pivot point. The RBD molecule has been omitted. (*B*) The EM structure reveals the presence of the two-up–one-down form of the spike protein. This is in contrast to the one-up–two-down form observed for all other nanobodies in the H11 class (Fig. 1*D*). (*C*) The hybrid nanobody appears to retain conformational flexibility by allowing interchange between up and down for one subunit.

The T99Y mutation in H11-H4 (introduced in B5 and B5-D4 hybrids) abolished binding (Table 3). In the H11-B5 complex, Y99 was accommodated by shifts in surrounding residues. We conclude that the additional compensating substitutions found in H11-B5 are required to allow these shifts. ITC shows that H11-H4 Q98R H100E (D4 hybrid) binds with a K_D_ of 18 nM to the isolated RBD with similar entropy and enthalpy as H11-H4 (Table 3 and Fig. 2). The crystal structure of the D4 hybrid bound to RBD reveals very little change of the main chain of the nanobody (RMSD of 0.5 Å over 125 Cα), except for a small shift that occurs in CDR1 at F29 (4 Å) (*SI Appendix*, Fig. S2*E*). The two mutated residues (Q98R and H100E) adopt the same conformation as they are found in the H11-D4 complex. Superimposing the hybrid RBD complex and H11-H4 RBD complex using only the atoms of RBD to calculate the superposition reveals that the hybrid has slightly pivoted on the surface of RBD, moving toward the orientation of H11-D4. The center of the motion is Y104 and can be described as a translation of 0.4 Å and rotation of 4° (Fig. 4*A*). As a result of the pivot, A14 (opposite end of the nanobody to Y104) has shifted by 3.6 Å. The hybrid has subtly altered its contacts with RBD including the π–cation interaction (Table 2).

The hybrid molecule was designed using the EM maps (the basis of the method) of the spike complexes, not the RBD complex crystal structures. H11-H4 and H11-D4 bind more weakly to the trimeric spike protein than to an isolated RBD molecule (29) (Table 4). The decrease in affinity arose despite the increase in enthalpy due to an increase in the entropic penalty (Table 4) (29). The hybrid molecule, in contrast to the parent, shows no loss of binding to the spike protein relative to RBD (Fig. 2 and Table 4). The data showed that although the enthalpy of binding to spike was reduced, the entropic penalty was also reduced, confirming the prediction from computational analysis. We determined the EM structure of the hybrid molecule with the spike. In contrast to the other structures which show a single one-up–two-down arrangement of the spike (Fig. 1*A*), the hybrid molecule is found in both one-up–two-down (80%) and two-up–one-down arrangements (20%) (Fig. 4*B*). We take this observation to indicate that in solution the arrangement of one of the RBD is dynamic, consistent with the decreased entropic binding penalty (Fig. 4*C*).

**Table 4.** Thermodynamic properties of selected nanobodies binding to spike

Nanobody	Thermodynamics (kJ/mol)							
	K_D_ (nM)	Change	ΔH	Change	TΔS	Change	ΔG	Change
H11-D4*	78 (±2)	+39↓	−81	−5↑	41	+7↓	−41	+1.7↓
H11-H4*	44 (±3)	+32↓	−74	−5↑	32	+9↓	−42	+3.3↓
H11-H4 Q98R H100E D4 hybrid	21 (±3)	+3→	−62	+7↓	18	−7↑	−44	0→

Arrows denote the following: ↑, an effect which improves binding compared to RBD; ↓, an effect which impairs binding; and →, an effect with no effect on binding.

^*^These data are from previous publications (29).

## Discussion

Although vaccination campaigns against SARS-CoV-2 have greatly reduced the number of deaths and the social disruption caused by COVID-19 (42–44), concern remains that future variants and escape mutants arising from monoclonal antibody treatment or waning immunity could trigger new waves of infections. Several animal studies have demonstrated the potency of nanobodies as therapies and prophylactics when administered by injection or topically (nasal) against SARS-CoV-2 (30, 45–48). Prior to COVID-19, a nanobody agent (49) delivered by inhalation against respiratory syncytial virus showed promise as a prophylactic and as an early-stage treatment (50–52).

Naïve library screening and maturation have generated multiple nanobodies that bound the spike protein of SARS-CoV-2 (Victoria strain) with micromolar to nanomolar affinity within a few weeks (29). As a result of binding to this epitope, the nanobody directly competes with the binding of angiotensin-converting enzyme 2 (ACE2) and thus is neutralizing (29). This epitope was subsequently termed cluster 2 (53) in one study of human antibodies and class 1/2 by a different study (54). Antibodies are known to bind to other portions of the spike protein, not just the RBD (55). A recent review (56) of known nanobody complexes with viral proteins revealed nanobodies primarily grouped into two distinct clusters (clusters 1 and 2). More recently, an in-depth experimental study of thousands of nanobodies has defined five binding classes defined by the recognition epitope surface (57). The binding affinity of nanobodies which compete with ACE2 binding was shown to correlate with neutralization potency (57) and represent a unique resource to investigate the structural determinants of affinity and thus inform the development of novel computational approaches.

Global measures of the interaction between these nanobodies and the RBD of the spike based on crystal structures with RBD failed to correlate with the observed biophysical data. At the heart of the interaction of these nanobodies with the RBD is a salt bridge π–cation interaction involving R52 from CDR2 of the nanobody and E484 and F490 of the virus RBD (Fig. 1*D*). The geometry of the interaction (distance and orientation) was correlated with the binding affinity, in that the more tightly binding nanobodies were associated with a more ideal geometry (Table 2). The combination of E484K (virus) and H11-H4 R52E showed no binding (Table 3 and *SI Appendix*, Fig. 6*C*), which we take to confirm the importance of precise geometry. This led us to the perhaps unexpected conclusion that the changes in CDR3 that were selected by phage display (Table 1) do not themselves increase the binding affinity by improving their specific interactions with RBD. Rather, the subtle sequence changes in CDR3 alter the nanobody in such a way as to optimize the CDR2 salt bridge π–cation interaction.

The observation that changes in residues distant from one site have profound influence at another is well known and commonly seen in directed evolution experiments where changes in nonactive site residues alter substrate specificity (58). This cooperative action at a distance often confounds simple modeling approaches. This is vividly illustrated in the case of the H11 nanobodies, where the sequence changes in CDR3 do not result in structural changes in CDR3 but do at CDR1 (which does not contact RBD and has no sequence change). These structural changes are then felt in a third part of the protein, CDR2, which improves its interaction with RBD. Individual site-directed mutagenesis experiments based on the crystal structures delineated contributions of specific residues, but we were unable to increase affinity by introducing mutations based on these insights (Table 3).

We examined the EM maps to identify global structural determinants of the binding affinity of the nanobody-spike complexes. The spike protein on its own is dynamic (59–61) but the binding of the H11 class of nanobodies apparently “freezes out” this motion consistent with the increased entropic penalty for binding. For H11-H4, the entropic penalty for freezing out the motion was estimated by comparing TΔS of binding to RBD and spike protein; this showed an increase of +9 kJ/mol (Table 4) (29), which would reduce affinity by over 20-fold. However, H11-H4 binds to the Spike improved enthalpy (ΔH −5 kJ/mol) analysis of the EM structure suggests the enthalpy gain comes from interactions between the nanobody bound to one monomer of the Spike with a neighboring monomer (29), these interactions not possible with the isolated RBD. As a result, the overall free energy of binding to spike compared to RBD has only reduced by 3 kJ/mol. We have previously shown that EM maps contain information about conformational dynamics that are typically not reported in the atomic coordinates of the final model (38–40). By analyzing the EM maps of the spike-nanobody complex, we were able to describe the conformational entropy of the spike-nanobody complexes. This is an attractive candidate to rationalize the binding affinity between the spike protein and nanobody. To test our understanding, we designed hybrids that would decrease the entropic penalty upon binding. We generated three hybrids (RTEYVSY, H4–D4 hybrid; RYEYVSY, H4–B5, D4 hybrid; and QYHYVSY, H4–B5 hybrid). Although this design approach lacks the sophistication of evolutionary selection, it did lead to hybrid nanobodies that exhibited improved binding affinity to the spike protein that did arise from a decrease in entropic penalty upon binding spike compared to RBD (TΔS 18 vs. 25 kJ/mol). The increase in the conformational variability (and therefore reduced entropic penalty) was clearly seen in the EM map of the complex. These results support that analysis of the conformational dynamics of EM maps yields actionable insights to improve the conformational entropy of binding.

## Conclusions

We have reported an analysis of the structural determinants of the binding affinity of a panel of nanobodies to the protein spike of SARS-CoV-2. Our results indicate that the overall conformational heterogeneity of a spike-nanobody complex is a strong determinant of the binding affinity. This finding identifies the binding entropy as an important contributor to the stability of spike-nanobody complexes and offers a design principle for the rational maturation of more potent nanobody variants.

## Methods

### Generation and Purification of Nanobodies and SARS-CoV-2 Proteins.

Detailed protocols have been described previously (29). In summary, a library of nanobodies was sourced from Abcore Inc. Phages displaying nanobodies (also known as variable heavy-chain domains of heavy-chain antibodies [VHHs]) specific for the SARS-CoV-2 RBD were enriched after two rounds of biopanning on 50 nM and 5 nM of RBD, respectively, through capturing with Dynabeads M-280 (Thermo Fisher Scientific). For each round of panning, the Dynabeads and phages were first blocked with StartingBlock Blocking Buffer (phosphate-buffered saline [PBS]; Thermo Fisher Scientific) for 30 min. The phages were incubated with the RBD for 1 h, then for 5 min with the Dynabeads (Thermo Fisher Scientific), and subsequently washed six times with PBS supplemented with 0.05% Tween-20 and once with PBS. The retained phages were eluted by incubation with Tris-buffered saline with added calcium chloride (TBS-C; 10 mM Tris, pH 7.4, 137 mM NaCl, and 1 mM CaCl_2_) and 1 mg/mL trypsin (Sigma-Aldrich) for 30 min. The collected phages were amplified in exponentially growing TG1 *Escherichia coli* cells and plated on 2xTY agar plates supplemented with 100 μg/mL ampicillin (2xTYA). Enrichment after each round of panning was determined by plating the cell culture with 10-fold serial dilutions. After the second round of panning, 93 individual clones were picked to inoculate 2xTYA and were grown overnight, with shaking at 250 rpm and 37 °C. The next day, the overnight culture was used to inoculate 2xTYA and infected with M13 helper phage to obtain clonal VHH-presenting phages. Nanobody H11 was selected, as it blocked ACE2 binding to RBD.

Affinity maturation of H11 was carried out by introducing random mutagenesis in the CDR3 region of nanobody H11 by PCR; the resulting PCR products were cloned into the pADL-23c phagemid (Antibody Design Laboratories). The ligated vector was transformed into TG1 cells by electroporation to give a phage library consisting of ∼2 × 10^9^ independent clones. Two rounds of biopanning of the library were carried out on 5 nM and 1 nM of RBD and positive phages were identified by enzyme-linked immunosorbent assay and sequenced, resulting in five further binders (Table 1).

H11-A10, H11-H6, H11-B5, H11-D4, and H11-H4 were cloned into the vector pOPINO containing an OmpA leader sequence and C-terminal His6 tag. For site-directed mutagenesis, the target VHH template was first amplified with two pairs of primers (as listed in Table 1): 1) PelB_Nb_Exp_F and a reverse primer containing the mutation and 2) a forward primer containing the mutation and PelB_Nb_Exp_R. Thereafter, the two fragments were included in one PCR and amplified with PelB_Nb_Exp_F and PelB_Nb_Exp_R. The resulting PCR product was cloned into the pADL-23c phagemid by Infusion cloning. The plasmids for H11-H4 T99Y, H11-H4 Q98R H100E, and H11-H4 Q98R T99Y H100E were generated through cloning “infusion-ready” gBlocks (Integrated DNA Technologies) into the pADL-23c phagemid. All constructs were verified by Sanger sequencing. The plasmids were transformed into the WK6 *E. coli* strain and protein expression was induced by 1 mM IPTG grown overnight at 28 °C. Periplasmic extracts were prepared by osmotic shock and VHH proteins were purified by immobilized metal affinity chromatography (IMAC) using an automated protocol implemented on an ÄKTXpress followed by a Hiload 16/60 Superdex 75 gel filtration column, using PBS buffer, pH 7.4.

Site-directed mutagenesis by PCR primer extension was employed to create six single DNA base substitution mutants of the best binder, H11-H4, and one double mutant of the parent nanobody, H11. H11-H4 and H11 in an ompA molecular cloning vector were used as DNA templates for their respective mutants (62). After the pADL-23c phagemid plasmid had been linearized at the desired insertion site, the ClonExpress II One Step Cloning Kit (Vazyme) was used to directionally insert the DNA fragment previously created into pADL-23c. The double and triple mutants of H11-H4 were ordered as synthetic genes. The variants generated are listed in Table 1.

Nanobodies were expressed and purified as described earlier (29). His-tagged SARS-CoV-2 RBD T332-K529 with Pro-527 omitted was expressed in expi293 cells and purified by immobilized metal affinity chromatography (IMAC), and size exclusion chromatography (SEC) was performed using a Superdex 75 HiLoad 16/600 gel filtration column. The production and purification of the Spike protein has been described before. Briefly, the gene encoding amino acids 1 to 1,208 of the SARS-CoV-2 spike glycoprotein ectodomain with mutations of RRAR > GSAS at residues 682 to 685 (the furin cleavage site) and KV > PP at residues 986 to 987, as well as inclusion of a T4 fibritin trimerization domain, was cloned into the pOPINTTGneo-BAP vector. Expi293 cells were cultured in expi293 expression media at 37 °C and 5% CO_2_ at 120 rpm for 17 h. The protein was purified by IMAC and SEC using a Superdex 200 HiLoad 16/600 gel filtration column (GE Healthcare).

### Structural Biology of the Nanobody Complexes.

Nanobodies were mixed with deglycosylated RBD at a molar ratio of 1.2:1, and the complex was purified by SEC similarly to H11-H4 and H11-D4 (29). The H11-H6 complex with RBD crystallized from a sitting drop (2:1 protein/reservoir) vapor diffusion against a reservoir of 0.1 M Hepes, pH 7.5, and 20% wt/vol polyethylene glycol (PEG) 8,000 at 18 mg/mL, while the H11-H4 Q98R H100E complex with RDB crystallized from a sitting drop (1:1 protein/reservoir) vapor diffusion against a reservoir of 0.1 M ammonium nitrate and 20% PEG 3,350 at 32 mg/mL In contrast to H11-D4, H11-H4, and H11-H6, we were unable to obtain well-diffracting crystals with RBD and the other three nanobodies. To aid crystallization, we added a second nanobody (F2) unrelated to the H11 class that bound to a different epitope. Using this approach, we obtained well-diffracting crystals of ternary complexes of RBD, F2 with H11 (reservoir 0.1 M BICINE, pH 8.5, and 20% wt/vol PEG 10,000), with H11-A10 (reservoir 0.1 M sodium citrate, pH 5.5, and 20% PEG 3,000), and with H11-B5 (reservoir 6% vol/vol Tacsimate, pH 6.0, 0.1 M MES monohydrate, pH 6.0, and 25% wt/vol PEG 4,000). All crystals were cryoprotected by the addition of 30% PEG 400, then flash cooled while mounted on a pin. Data were collected at beamline I03 at Diamond Light Source. Crystal structures were solved by molecular replacement with PHASER (63) implemented in CCP4 (64) using the RBD and H11-H4 from RCSB Protein Data Bank (PDB) accession 6ZBP (29). Structures were refined in REFMAC5 (65) aided by PDB-REDO (66), MOLPROBITY (67), and the TLSMD server (68). Statistics for X-ray data collection and structure refinement are given in *SI Appendix*, Table S1.

In a similar manner reported for the H11-H4 complex (29), spike-nanobody complexes were mixed at a 1:1.2 molar ratio and incubated at 16 °C overnight. Approximately 6 nL was applied to glow-discharged grids (Harrick Plasma Cleaner PDC-002-CE) with the Chameleon EP system (SPT Labtech) at 79 to 83% relative humidity and ambient temperature. Spike-NB H11-H4 Q98R H100E at 1 mg/mL was applied to Quantifoil 200-mesh Au R1.2/1.3 grids, glow-discharged at 30 mA twice for 1 min (Quorum GloQube), and plunge-frozen using a Vitrobot MarkIV. EM data were collected with EPU on Titan G2 microscopes (Thermo Fisher Scientific) equipped with a Bioquatum-K3 detector (Gatan) operated at 300 kV using an energy-selecting slit of 20 eV (*SI Appendix*, Table S2). Data were collected on Krios II and IV located at the electron Bio-Imaging Centre (eBIC) at Diamond Light Source (under proposals BI27051 and BI29666). Processing up to two-dimensional classification used the Relion_IT.py processing pipeline implemented at eBIC at Diamond Light Source. Data were processed in C1 for further refinement, CTF refinement, and particle polishing within Relion. Data processing and refinement statistics are given in *SI Appendix*, Table S2 and started from the H11-H4 Spike structure PDB accession 6ZHD (29). The high-resolution crystal structures of RBD-nanobody were used to fit the nanobody as a rigid body, as the density for the nanobodies was quite poorly resolved. The final models were obtained by multiple rounds of jelly body refinement using RefMac5 via CCP-EM GUI (65, 69) or Phenix real space refinement (70) and manual intervention with coot (71).

### Biophysical Analysis of the Nanobodies.

Biophysical analysis followed previous reports (29). Briefly, SPR experiments were performed using a Biacore T200 (GE Healthcare) in PBS, pH 7.4, supplemented with 0.005% vol/vol surfactant P20 (GE Healthcare) at 25 °C. Binding experiments of H11, H11-A10, H11-H6, H11-B5, H11-D4, and H11-H4 were performed using a Sensor Chip Protein A (Cytiva) with RBD-Fc immobilized. Other binding experiments performed using a Biotin CAPture Kit (Cytiva) with biotinylated RBD immobilized. Nanobodies were injected with serial two-fold dilutions. Background correction was carried out by buffer-only injection. Data were fitted to a 1:1 binding model using Biacore T200 Evaluation Software 3.1. For the binding of H4_R52A/E/K mutants to the RBDs, a single injection of the nanobodies at 1 μM was performed over the biotinylated RBDs. ITC measurements were carried out using an iTC200 MicroCalorimeter (GE Healthcare) at 25 °C. RBD and all nanobodies were prepared and dialyzed in PBS. Nanobodies were titrated into RBD solution as a 0.4 μL initial injection with a further 16 injections of 2.4 μL made under stirring at 750 rpm. Data acquisition and analysis were performed using the Origin scientific graphing and analysis software package (OriginLab). Data analysis was performed by generating a binding isotherm and best fit using the following parameters: N (number of sites), ΔH (in kJ/mol), ΔS (in J mol^−1^ K^−1^), and K (binding constant in moles). Following data analysis, K was converted to the dissociation constant (K_D_).

Thermal stability assays were performed using a NanoTemper Prometheus NT.48. Eleven microliters of protein at ∼1 mg/mL (some concentrations were adjusted slightly to give optimal signal) was loaded into a capillary and heated from 15 to 95 °C at a rate of 1 °C per minute. Three repeats were made for each of the nanobodies. Analysis was performed using PR.ThermControl v2.3.1 software.

### Computational Analysis of the Binding Affinity of the Nanobodies.

EMMI is a Bayesian approach to model statistical ensembles by combining prior information on a system with experimental data subject to noise or systematic errors (38–40). This method is optimally suited for determining structural ensemble through molecular dynamics simulations, in which the prior (i.e., the force field) is corrected by information from experimental cryo-EM data. To be able to compare microscopic structures with a cryo-EM data voxel map, one has to devise a forward model. In EMMI, the cryo-EM data voxel map at position ***x*** is represented as a Gaussian mixture model (GMM) *ϕ_D_(****x****)* with *N_D_* components (data GMM) as follows:

$$\phi_{D}\left(x\right)=\overset{N_{D}}{\underset{i=1}{\sum }}\phi_{D,i\;}\left(x\right)=\overset{N_{D}}{\underset{i=1}{\sum }}\omega_{D,i}G(x\;\text{|}x_{D,i},\Sigma_{D,i}),$$

where each *i*th component of the data GMM weights by *ω_D,i_* and a normalized Gaussian function ***G*** is centered in ***x****_D,i_* with a corresponding covariance matrix **Σ***_D,i_* (38). EMMI measures the deviation between the GMMs generated from experimental data and molecular dynamics by utilizing the overlap function:

$$o_{MD,i}=\int^{\;}dx\;\phi_{M}\;\left(x\right)\phi_{D,i}\left(x\right),$$

where *ϕ_M_(****x****)* represents the forward model GMM, in which each heavy atom of the system is modeled by a Gaussian function (38). To deal with the heterogeneity of the system, EMMI simulates many replicas of it, and the overlap between the model and the data GMM is estimated over the ensemble of replicas, yielding an average overlap ${\bar{o}}_{MD,i}$. Finally, usually the error in the data are sampled a posteriori, thereby simplifying the total energy function to the following (72):

$$E_{\mathrm{EMMI}}=\;E_{MD}+\;\frac{k_{B}T}{2}\;\underset{r,i}{\sum }\log\left[\frac{1}{2\;\left(o_{DD,i}-{\bar{o}}_{MD,i}\right)}\;\mathrm{erf}\left(\frac{o_{DD,i}-{\bar{o}}_{MD,i}}{\sqrt{2}\sigma_{r,i}^{\mathrm{SEM}}}\right)\right]\;,$$

where the first term represents the forcefield energy and the second term quantifies an energy penalty depending on the agreement of the cryo-EM data GMM with the molecular dynamics generated models (38).

The initial structure of the spike was constructed as in ref. 73 except for the case of the H11-H4 Q98R H100E D4 hybrid system, which has two RBDs in the open state (2UP-RBD). The 2UP-RBD conformation recapitulates a previously determined atomic model of spike protein bound to the neutralizing nanobody Nb12 (PDB accession 7MY3), the initial structure for the 2UP-RBD structure was constructed using 7MY3 as a template. From here, missing residues from 7MY3 were added using homology modeling as in ref. 73 and with guidance from the associated cryo-EM density maps (Electron Microscopy Data Bank [EMD] accessions EMD-14576 and EMD-14544). Refinement of backbone and side-chain angle outliers was performed using the ChimeraX tool ISOLDE (74).

The initial structures of the nanobodies were obtained from the following PDB accessions: 6ZHD (H11-H4), 6Z43 (H11-D4), 7Z1D (H11-H6), 7Z1C (H11-B5), 7Z1A (H11), and 7Z1E (H11-D4 Q98RH100E 1UP). Then, for each complex, the spike and nanobody were docked and aligned to the respective cryo-EM map (EMD accessions EMD-11218, EMD-11068, EMD-14539, EMD-14543, EMD-14531, EMD-14576, and EMD-14544). In this manner, the missing loops and glycans on the spike are taken into account.

For the following steps, the nanobodies with the highest affinity to the spike were selected. For systems H11-H4, H11-D4, H11-H6, H11-B5, H11, H11-H4 Q98RH100E D4 hybrid 1UP, and H11-H4 Q98RH100E D4 hybrid 2UP, we proceeded by setting up a simulation box comprising 583088, 584204, 581678, 574397, 559778, 600449, 617361 atoms, respectively. The protein, water, and glycan force fields employed in this study are AMBER99SB-ildn (75), TIP3P (76), and GLYCAM06h (77). The system was subsequently energy minimized, equilibrated in an NPT and subsequent NVT molecular dynamics equilibration using GROMACS-2018.6 (78). To constrain bond lengths, we used the LINCS algorithm (79). The Lennard–Jones interactions are treated with a 1-nm cutoff, while the electrostatic interactions are treated with the particle-mesh Ewald method using a Fourier spacing of 1.2 nm and a 1-nm cutoff for the short-range electrostatic part. Pair-lists are updated every 10 fs, using a cutoff of 1 nm and a time step of 2 fs (75). Integration of Newton’s equations of motion was performed using the leap-frog algorithm, the velocity-rescaling thermostat (80) with a coupling time constant of 0.2 ps, and the Parrinello-Rahman barostat (81) for equilibration utilizing a coupling time constant of 1.0 ps during NPT simulations. In the NPT equilibration, positions of the Cα atoms were restrained with a constant force of 200 kJ/mol/nm^2^, and the temperature was set to 310 K, pressure to 1 atm, and simulation duration to 500 ps. In the NVT equilibration, we lifted the position restraints, simulated for 2 ns, and set the temperature to 310 K without pressure coupling.

The experimental voxel map data for complexes H11-H4, H11-D4, H11-H6, H11-B5, H11, H11-H4 Q98RH100E D4 hybrid (1UP), and H11-H4 Q98RH100E D4 hybrid (2UP) are expressed as a data GMM containing 10,000 Gaussians each, exhibiting a correlation of 0.98, 0.87, 0.98, 0.98, 0.8, 0.83, and 0.93 with the original experimental voxel map. We extracted 32 configurations from the previous NVT equilibration and initiated two individual EMMI simulations, each consisting of 32 replicas with an aggregate runtime of 1 μs using PLUMED.2.6.0-dev (82). EMMI simulations were performed in the NVT ensemble using the same molecular dynamics parameters as in the equilibration step. Configurations were saved every 5 ps for postprocessing. The cryo-EM restraint is calculated every two steps, using neighbor lists to compute the overlaps between the model and data GMMs, with a cutoff equal to 0.01 and an update frequency of 100 steps.

For each EMMI simulation, we removed the first 2 ns, divided the remaining trajectory into two blocks of equal length, and performed cluster analysis using Gromos clustering with a cutoff of 3 Å and considering spike epitope residues 446 to 458 and 471 to 496 as well as CDR residues 29 to 32, 52 to 57, and 98 to 108. We identified the populations of each cluster in each block and the SD. Such block averages and SDs represent a measure of convergence of our simulations (*SI Appendix*, Fig. S1). For each system, we constructed the free energy surface along the RMSDs from its most populated cluster. This free energy surface quantifies the effect of the dynamics on the complex. For molecular visualizations, we used visual molecular dynamics (83).

## Supplementary Material

Supplementary File

## Data Availability

The coordinates and structure factors were deposited in PDB under the following accession numbers: H11 and F2 bound to RBD (7Z1A) (84), H11-A10 and F2 bound to RBD (7Z1B) (85), H11-B5 and F2 bound to RBD (7Z1C) (86), H11-H6 bound to RBD (7Z1D) (87), and H11-H4 Q98R H100E D4 hybrid bound to RBD (7Z1E) (88). EM structures and maps are available in PDB and EMD under the following accession numbers, respectively: Spike bound H11 (7Z6V and EMD-14531) (89, 90), H11-H6 (7Z7X and EMD-14539) (91, 92), H11-A10 (7Z9Q and EMD-14575) (93, 94), H11-B5 (7Z85 and EMD-14543) (95, 96), and H11-H4 Q98R H100E D4 hybrid (one-up–two-down: 7Z86 and EMD-14544; and two-up–one-down: 7Z9R and EMD-14576) (97–100). The following plasmids are deposited with Addgene under the following accession numbers: 184277 (pOPINO_H11), 184278 (pOPINOH11-A10), 184279 (pOPINO_H11-B5), 184280 (pOPINO_11-H6), and 184281 (pOPINH11-H4 and Q98R_H100E). All other study data are included in the article and/or *SI Appendix*.
