# Supplementary material for: Correlation between the binding affinity and the conformational entropy of nanobody SARS-CoV-2 spike protein complexes
Source: Proc Natl Acad Sci U S A. 2022 Jul 15;119(31):e2205412119. doi: 10.1073/pnas.2205412119 (PMC9351521; doi:10.1073/pnas.2205412119)
Supplement: Supplementary File [file pnas.2205412119.sapp.pdf]

Correlation between binding affinity and conformational entropy of nanobodies targeting the SARS-CoV-2 spike protein

Supporting Online Information

Figures 1 to 6
Tables 1 to 6
References

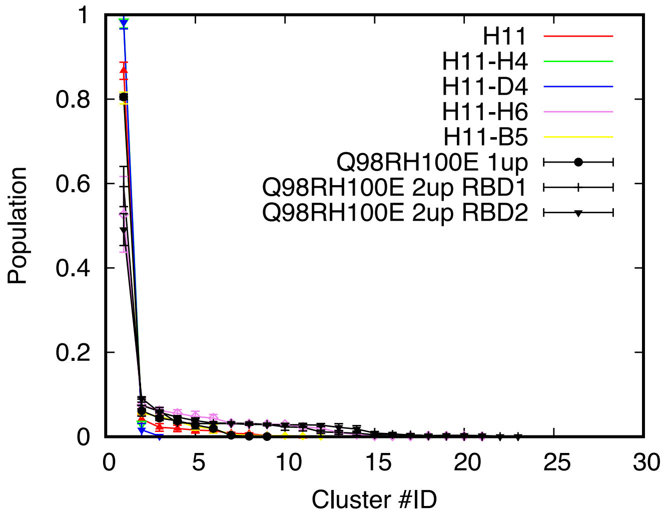

Figure S1. Analysis of the structural ensembles of the spike-nanobody complexes determined in this study. Convergence analysis of the structural ensembles, showing the populations of structural states for the RBD-nanobody complexes of the various systems.

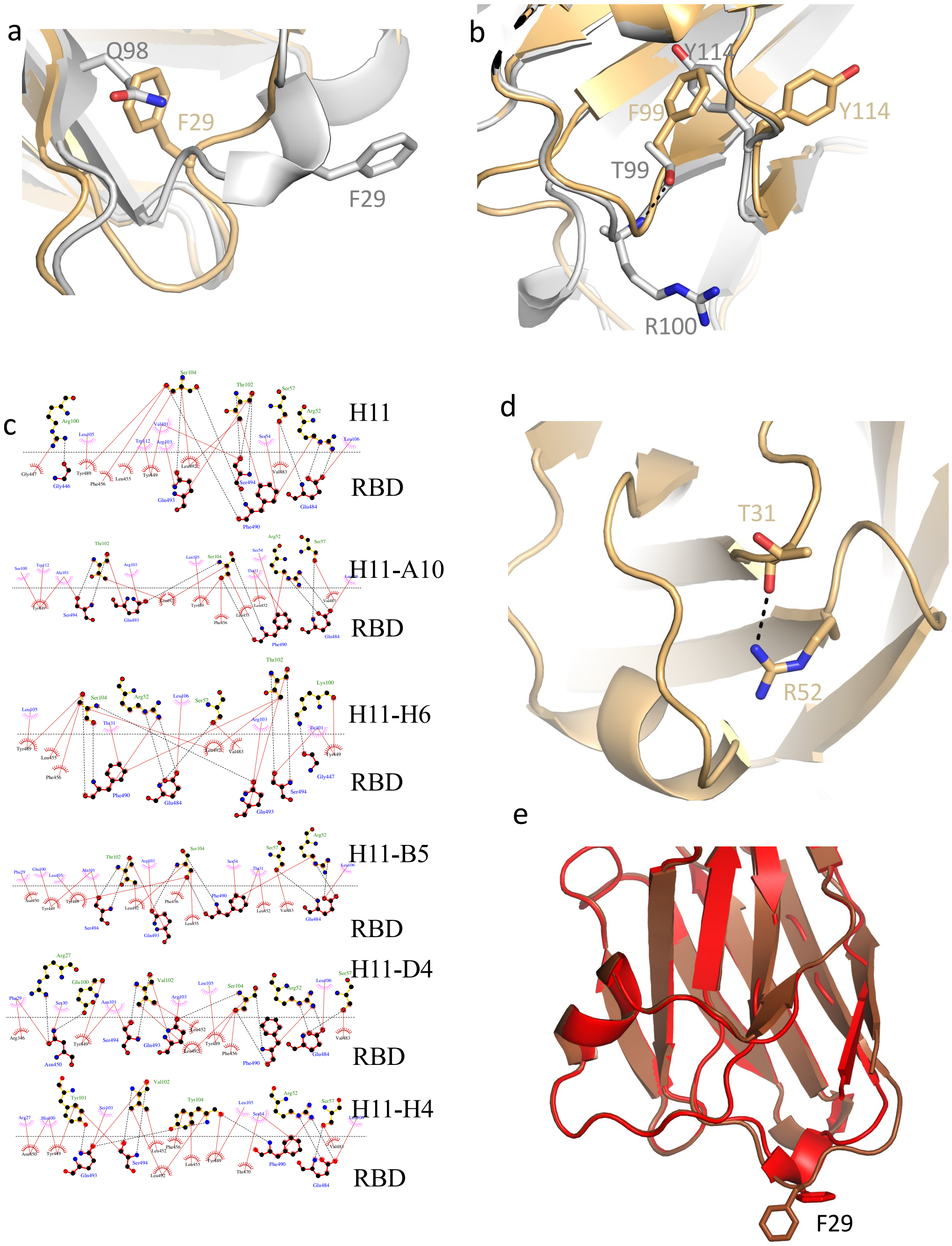

Figure S2. Detailed structural interactions between the nanobodies and RBD. **(a)** The change from Q98 in H11 (in grey) to smaller side chains (G98) in H11-A10 (shown in wheat), H11-B5 (S98) and in H11-H6 (G98) creates a void which is filled by F29. The structure of CDR1 is altered but CDR3 is preserved. G98 is not labelled, other side chains are labelled with color matching the nanobody. **(b)** The change from T99 in H11 (in grey) to bulky aromatic side chains (F99) in H11-A10 (wheat) and H11-B5 (Y99) displaces Y114. The change from threonine (or serine) also removes the hydrogen bond to the amide of residue 100. nanobodies interactions with the RBD. The data from H11-D4 and H11-H4 have been previously reported(1). (c) Ligplot(2) analysis of the nanobodies' interactions with RBD derived from the crystal structure. The data from H11-D4 and H11-H4 have been previously reported(1); water molecules have been omitted for clarity. **(d)** In H11-A10 (wheat), H11-B5 and H11-H6 T31 hydrogen bonds to the key R52. The change of CDR1 in H11, H11-D4 and H11-H4 do not permit this hydrogen bond (1). H11-D4 and H11-H4 have a water molecule bound to R52. In the H11structure no water is seen perhaps due to its lower resolution. **(e)** The D4 hybrid (brown) overlays closely with H11-H4, there is a significant shift at F29.

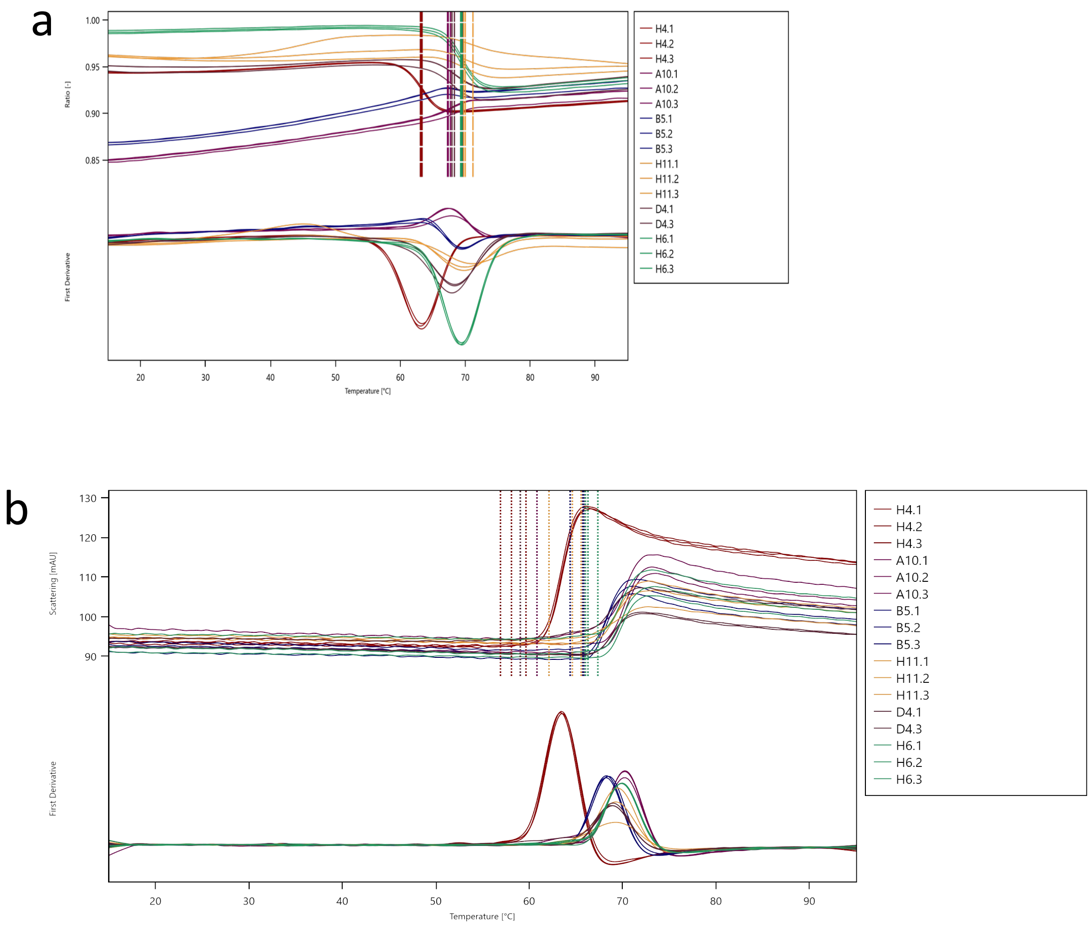

Figure S3. Thermal denaturation analysis of the nanobodies. **(a)**. Fluorescence ratio at 350 nm/330 nm and first derivative. Three repeats were made for each nanobodies. No major significant changes of the Tm is seen for the H11 series except for H4. **(b)**. Light scattering of the nanobodies and first derivative. More significant aggregation can be detected for H4.

a

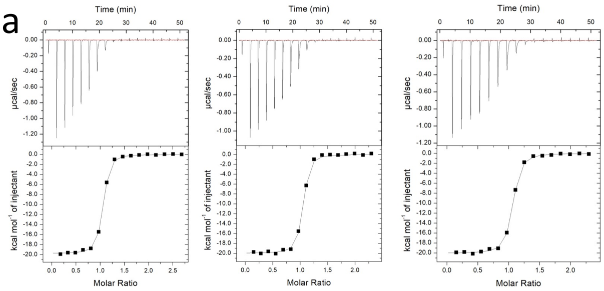

RBD (cell)
H11-A10 (syringe)
N=1
ΔH= -83 kJ mol^{-1}
ΔG= -41 J mol^{-1}/K^{-1}

b

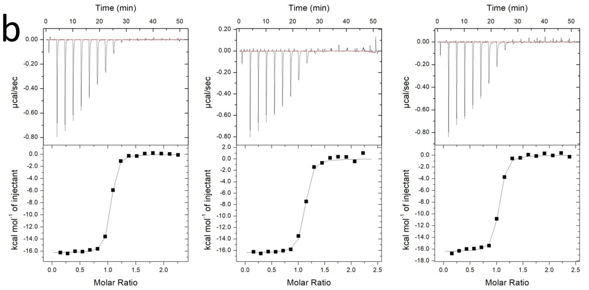

RBD (cell)
H11-B5 (syringe)
N=1
ΔH= -68 kJ mol^{-1}
ΔG= -41 J mol^{-1}/K^{-1}

c

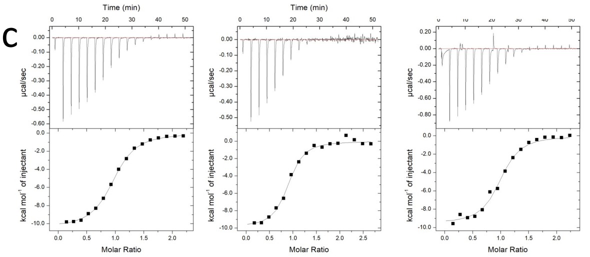

RBD (cell)
H4 Y101A (syringe)
N=1
ΔH= -42 kJ mol^{-1}
ΔG= -34 J mol^{-1}/K^{-1}

d

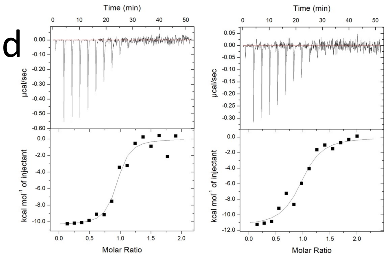

RBD (cell)
H4 Y104S (syringe)
N=1
ΔH= -46 kJ mol^{-1}
ΔG= -37 J mol^{-1}/K^{-1}

e

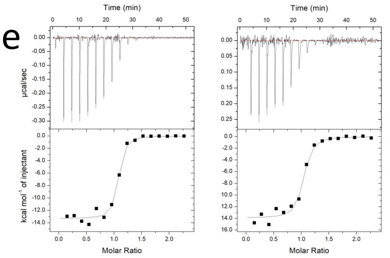

RBD (cell)
H4 Y104F (syringe)
N=1
ΔH= -57 kJ mol^{-1}
ΔG= -42 J mol^{-1}/K^{-1}

f

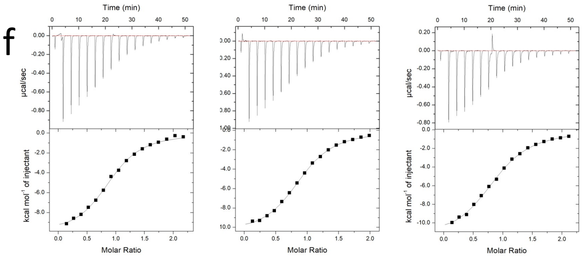

RBD (cell)
H4 V101Y R103S (syringe)
N=1
ΔH= -46 kJ mol^{-1}
ΔG= -30 J mol^{-1}/K^{-1}

g

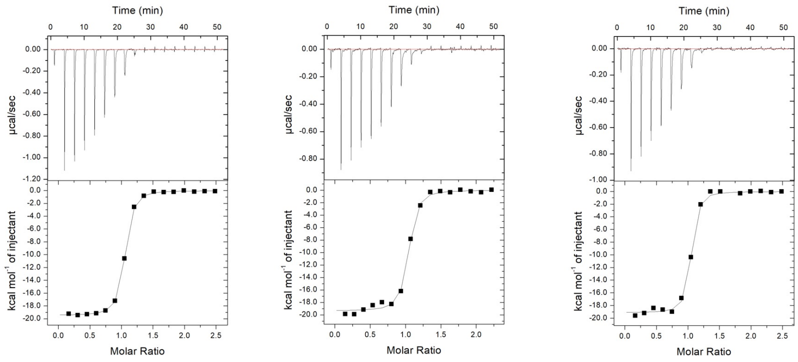

RBD (cell)
H11-H6 (syringe)
N=1
ΔH= -80 kJ mol^{-1}
ΔG= -41 J mol^{-1}/K^{-1}

h

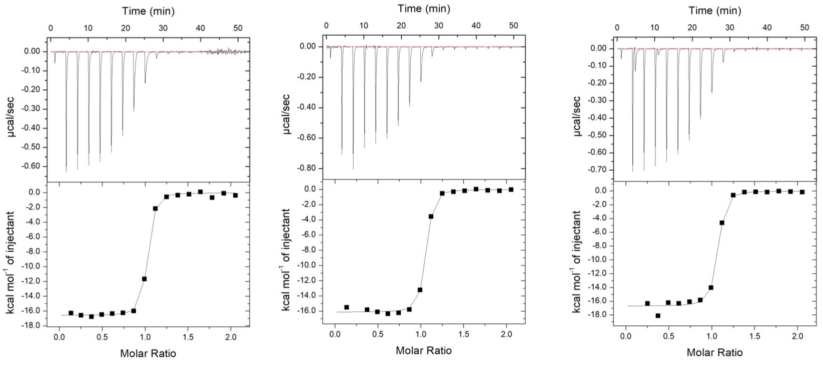

RBD (cell)
H11-H4 Q98R H100E (syringe)
N=1
ΔH= -69 kJ mol^{-1}
ΔG= -44 J mol^{-1}/K^{-1}

i

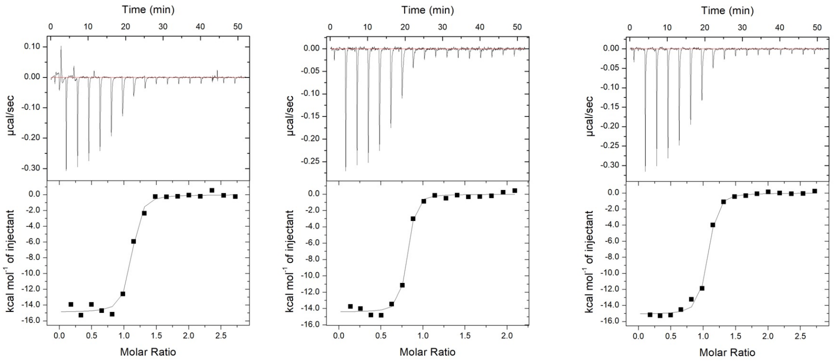

Spike (cell)
H11-H4 Q98R H100E (syringe)
N=1
ΔH= -62 kJ mol^{-1}
ΔG= -44 J mol^{-1}/K^{-1}

Figure S4. Analysis of nanobody binding by Isothermal calorimetry (a) Titration of the H11-A10 nanobody (200 µM) into RBD (18 µM) (three independent measurements), **(b)** Titration of the H11-B5 nanobody (220 µM) into RBD (19 µM) (three independent measurements), **(c)** Titration of the H4 Y104A nanobody (290 µM) into RBD (29 µM) (three independent measurements), the very tight binding make fitting thermodynamic parameters prone to large error, **(d)** Titration of the H4 Y104S nanobody (280 µM) into RBD (28 µM) (two independent measurements) **(e)** Titration of the H4 Y104F nanobody (147 µM) into RBD (15 µM) (two independent measurements) **(f)** Titration of the H4 V101Y + R103S nanobody (477 µM) into RBD (46 µM) (three independent measurements)) **(g)** Titration of the H6 nanobody (200 µM) into RBD (21 µM) (three independent measurements). **(h)** Titration of the H4 Q98R H100E nanobody (170 µM) into RBD (17 µM) (three independent measurements). **(i)** Titration of the H4 Q98R H100E nanobody (82 µM) into spike (8 µM) (three independent measurements). Other raw data have previously been published (1, 3).

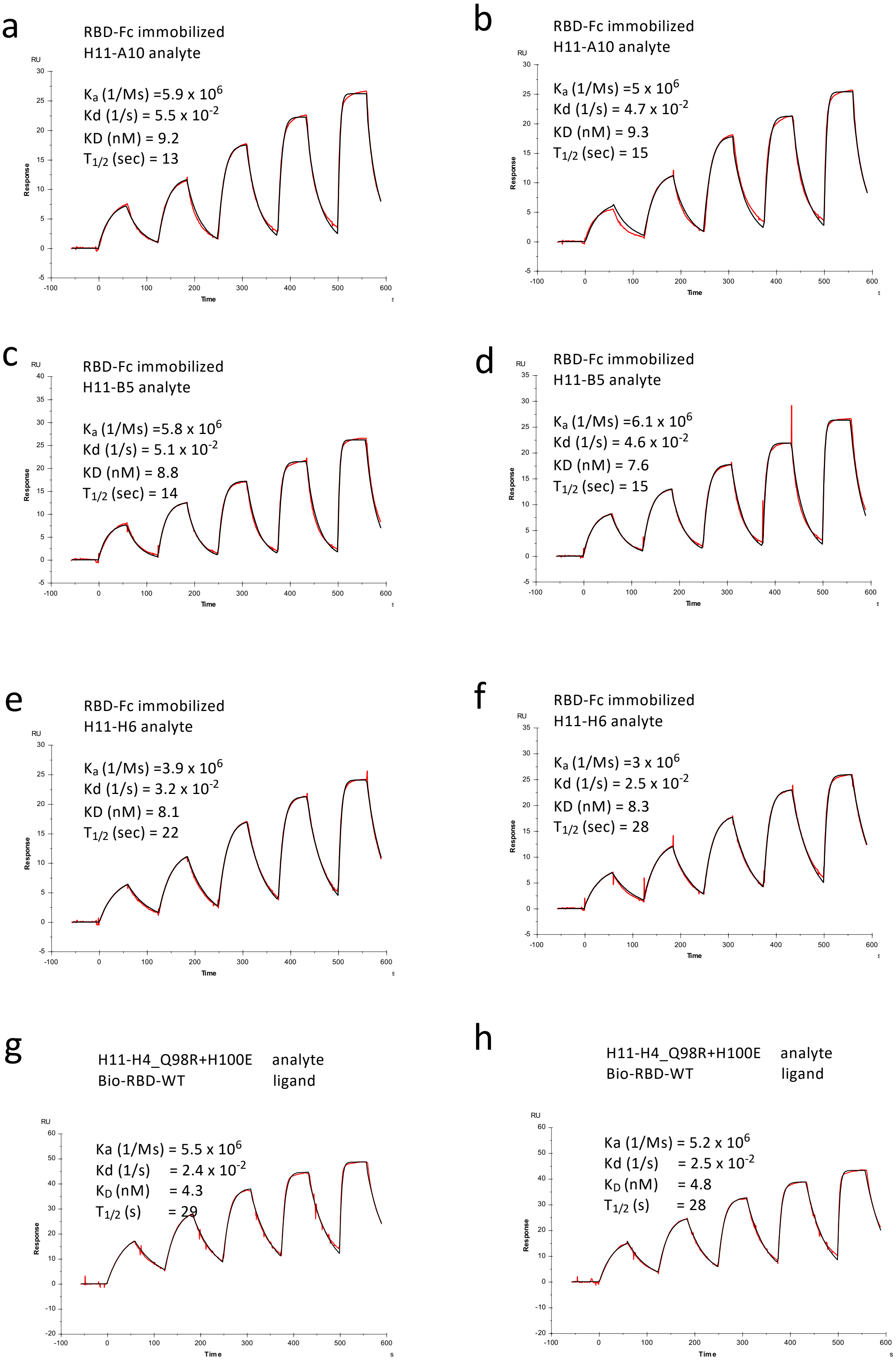

Figure S5. Nanobody binding kinetics SPR replicated sensorgrams for **(a, b)** H11-A10, **(c, d)** H11-B5, (**e, f**) H11-H6 and (**g, h**) H11-H4-Q98RH110E D4 hydrid, binding to RBD (immobilized as RBD-Fc on the chip).

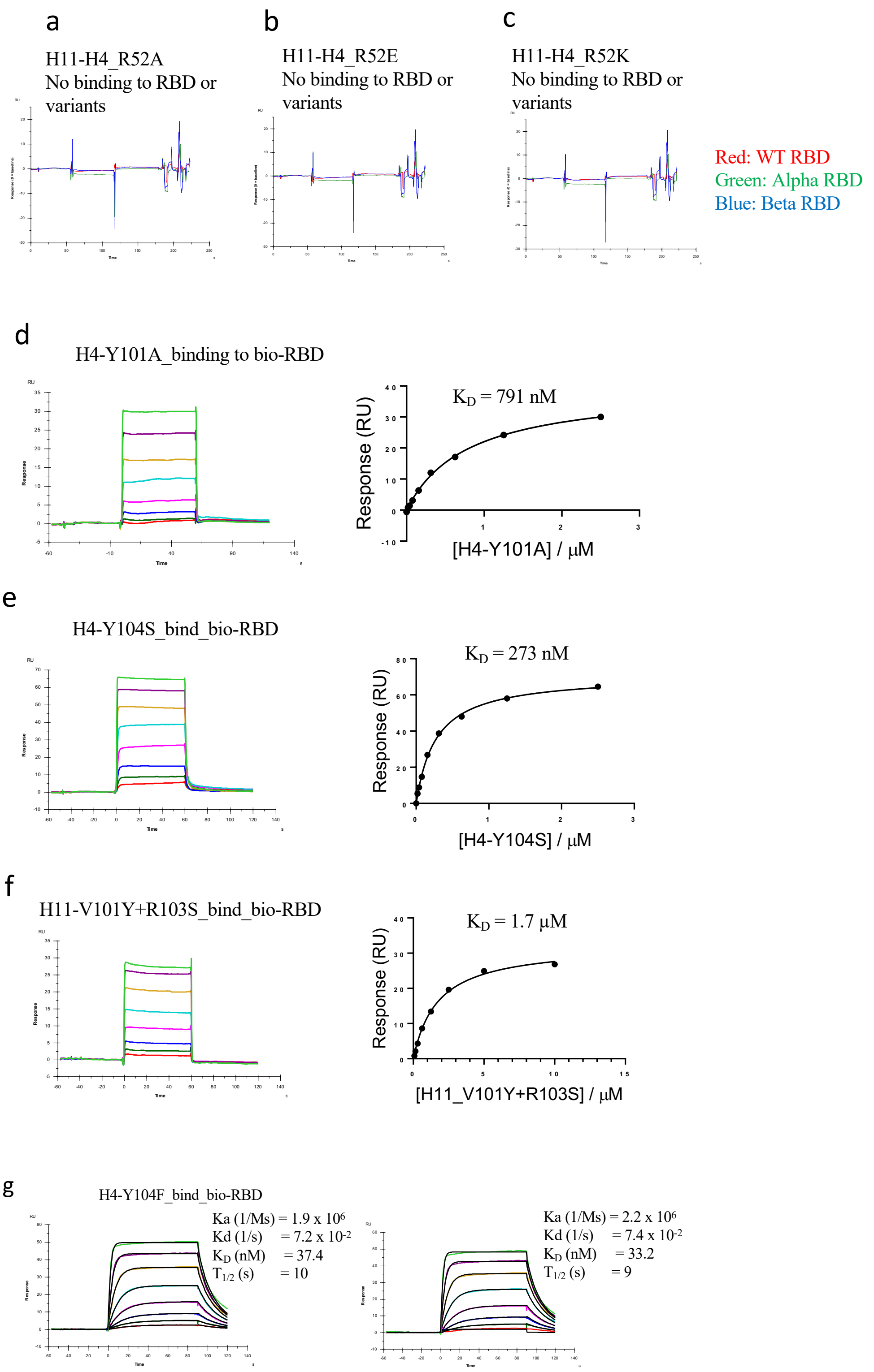

Supporting Information Figure S6. (a,b & c) Mutation of R52 in the nanobody abolishes binding to RBD from alpha, beta and delta strains of SARS-CoV)-2. Nanobody binding kinetics. (d) SPR data and estimated binding constant of H11-H4 Y101A. (e) SPR data and estimated binding constant of H11-H4 Y101S. (f) SPR data and estimated binding constant of H11-H4 YR03S. (g) SPR data and estimated binding constant of H11-H4 Y104F.

Table S1. Crystallography and data collection and refinement statistics, nanobody complexes.

	H11-F2-RBD (PDB 7Z1A)	H11-A10-F2-RBD (PDB 7Z1B)	H11-B5–F2-RBD (PDB 7Z1C)	H11-H6–RBD (PDB 7Z1D)	(H11-H4 Q98R H100E D4 hybrid) (PDB 7Z1E)
**Data collection**					
Space group	$P2_12_12$	$P2_12_12_1$	$P\,1$	$P2_12_12_1$	$P\,3_121$
Cell dimensions					
a, *b*, *c* (Å)	157, 191.6, 56.8	58.5, 120.8, 171.9	56.9, 56.9, 115.9	62.4, 65.1, 82.9	78.2, 78.2, 126.8
α, β, γ (°)	90, 90, 90	90, 90, 90	80, 87.5, 66.1	90, 90, 90	90, 90, 120
Resolution (Å)[a]	59–2.59 (2.66– 2.59)	57-2.3 (2.34–2.3)	52–1.90 (1.95–1.90)	45–1.55 (1.59-1.55)	67.7–1.59 (1.62–1.59)
R_{merge}	0.125 (4.0)	0.129 (2.1)	0.04 (1.1)	0.08 (1.2)	0.04 (4.0)
R_{pim}	0.05 (1.6)	0.05 (0.92)	0.04 (1.1)	0.08 (1.3)	0.01 (1.3)
$I/\sigma(I)$	14.6 (0.7)	12 (1.2)	10 (0.8)	18.3 (2.2)	24.9 (0.4)
$CC_{1/2}$	1.0 (0.3)	1.0 (0.5)	1.0 (0.5)	1.0 (0.7)	1.0 (0.4)
Completeness (%)	100 (100)	100 (100)	96.4 (91)	98.5 (97)	100 (100)
Measurements	54473 (2641)	55151 (2742)	99469 (4623)	48875 (2350)	61104 (3027)
Wilson B	66	49	39	18	33
Redundancy	13.6 (14.3)	13.4 (12.3)	3.6 (3.2)	13.5 (13.2)	18.8 (20.2)
**Refinement**					
Resolution (Å)	59.16–2.59 (2.65–2.59)	57–2.30 (2.36–2.30)	52–1.90 (1.95–1.90)	45.09–1.55 (1.59–1.55)	67.76–1.59 (1.63–1.59)
No. reflections	54473 (3936)	55151 (4029)	99469 (6919)	48875(3509)	61035(4228)
R_{work} / R_{free}	20.8 / 24.1 (44.5 / 42.6)	19.7 / 24.3 (34.4 / 34.6)	17.5 / 19.9 (37.1/ 37.4)	16.5 / 19.1 (25.2/ 28)	16.1 / 20.2 (45.7 / 51.1)
No. atoms					
Protein	7070	7148	7046	2563	2574
Ions / buffer	28	28	84	20	26
Water	9	153	339	312	205
Residual *B* factors					
Protein	60	11	32	23	42
Ligand/ion	104	95	75	36	52
Water	81	59	58	33	48
Ramachandran					
Favored (%)	96.2	96.5	97.6	98.9	98.4
Disallowed (%)	0.3	0.8	0	0	0.3
R.m.s. deviations					
Bond lengths (Å)	0.008	0.008	0.007	0.013	0.005
Bond angles (°)	1.61	1.49	1.39	1.71	1.31

Data were collected from a single crystal for each structure.

[a] Values in parentheses are for highest-resolution shell.

Table S2. Cryo-EM data collection parameters and refinement statistics.

	Spike H11 (PDB 7Z6V EMD-14531)	Spike H11-H6 (PDB 7Z7X EMD-14539)	Spike H11-A10 (PDB 7Z9Q EMD-14575)	Spike H11-B5 (PDB 7Z85 EMD-14543)	Spike H11-H4 Q98R H100E, 1up2down (PDB 7Z86 EMD-14544)	Spike H11-H4 Q98R H100E D4 hybrid, 2up1down (PDB 7Z9R EMD-14576)
**Data collection and processing**						
Magnification	81,000	81,000	81000	81,000	81,000	81,000
Voltage (kV)	300	300	300	300	300	300
Electron exposure ($e^-/Å^2$)	46.5	50	51.5	45	50	50
Defocus range (μm)	1.5-3.0	1.5-3.0	1.5-3.0	1.5-3.0	1.5 – 3.0	1.5 – 3.0
Pixel size (Å/pix) (Super resolution)	0.53	0.53	0.53	0.53	0.53	0.53
Symmetry imposed	*C*1	*C*1	*C*1	*C*1	*C*1	*C*1
Initial particle images (no.)	911,269	1,609,112	3,264,123	672,533	909,790	909,790
Final particle images (no.)	432,130	210,571	305,820	318,816	396,910	110,410
Map resolution (Å)	3.1	3.3	3.6	3.1	3.4	4.2
FSC threshold	0.143	0.143	0.143	0.143	0.143	0.143
Map resolution range (Å)						
**Refinement**[a]						
Initial model used	6ZHD	6ZHD	6ZHD	6ZHD	6ZHD	6ZHD
Model resolution (Å)	3.0	2.9	3.1	2.8	3.2	3.6
FSC threshold	0.143	0.143	0.143	0.143	0.143	0.143
Model resolution range (Å)	3.1-3.0	3.3-2.8	3.6-3.0	3.5-2.6	3.4-3.0	4.1-3.6
Map sharpening *B* factor ($Å^2$)	-97	-118	-130	-126	-182	-166
Model composition						
Non-hydrogen atoms	27,154	26,906	27,065	26,834	27,071	27,078
Protein residues	3,360	3,353	3,371	3,344	3348	3349
B factors ($Å^2$)						
Protein	147	90	340	132	241	228
R.m.s. deviations						
Bond lengths (Å)	0.009	0.006	0.009	0.007	0.011	0.008
Bond angles (°)	1.66	0.668	1.55	0.835	1.34	1.41
**Validation**						
MolProbity score	1.6	1.74	1.5	1.5	1.33	1.61
Clashscore	7.9	8.04	4.6	5.5	4.1	6
Poor rotamers (%)	0.45	0.1	0.9	1.0	0.76	1.2
Ramachandran plot						
Favored (%)	97.1	95.7	96.3	96.4	97.2	96.6
Allowed (%)	2.8	4.3	3.4	3.6	2.8	3.4
Disallowed (%)	0.1	0	0.3	0	0.0	0.0

Table S3. Superposition of the crystal structures of the nanobody RBD complex

	H11	H11-A10	H11-B5	H11-H6	H11-D4	H11-H4
**NANO**[1]						
H11	-	-	-	-	-	
H11-A10	0.83 (126)	-	-	-	-	
H11-B5	0.53 (122)	0.57 (125)	-	-	-	
H11-H6	0.85 (126)	0.43 (127)	0.69 (125)	-	-	
H11-D4	0.60 (127)	0.56 (124)	0.60 (122)	0.59 (124)	-	
H11-H4	0.50 (128)	0.95 (127)	0.58 (122)	0.93 (126)	0.40 (126)	-
H11-H4-D4-hybrid	0.60 (125)	0.52 (121)	0.73 (122)	0.52 (121)	0.54 (125)	0.56 (125)
**RBD**[2]						
H11	-	-	-	-	-	
H11-A10	0.56 (196)	-	-	-	-	
H11-B5	0.31 (196)	0.42 (196)	-	-	-	
H11-H6	0.59 (194)	0.71 (193)	0.62 (194)	-	-	
H11-D4	0.96 (195)	0.96 (194)	0.94 (194)	0.72 (194)	-	
H11-H4	0.87 (195)	0.98 (194)	0.87 (194)	0.64 (194)	0.51 (195)	-
H11-H4-D4-hybird	0.74 (193)	0.81 (194)	0.80 (194)	0.56 (194)	0.39 (194)	0.43 (194)

[1] Superposition and rmsd for the nanobody (Å)

[2] Superposition and rmsd for the RBD (Å)

Table S4. Analysis of the ratio of B-factor of the loops with the average B factor for the nanobody and the melting temperature for the nanobodies.

	**CDR1 (29-32)**	**CDR2 (52-57)**	**CDR3 (98-108)**	**TM °C**
H11	0.90	0.86	0.75	70 ± 0.7
H11-A10	0.91	0.89	0.76	68 ± 0.3
H11-B5	0.97	0.83	0.65	70 ± 0.1
H11-H6	0.93	0.86	0.76	69 ± 0.1
H11-D4	0.91	0.73	0.67	68 ± 0.3
H11-H4	0.86	0.73	0.79	63 ± 0.1

Table S5. SPR data from **Figure S5**

**Analyte**	**ka (1/Ms)**	**kd (1/s)**	**K_D (nM)**	**$T_{1/2}$ (s)**	**Average $T_{1/2}$ (s)**
H11-A10	5.9E+06	5.5E-02	9.2	13	14
H11-A10	5.0E+06	4.7E-02	9.3	15	
H11-B5	5.8E+06	5.1E-02	8.8	14	14
H11-B5	6.1E+06	4.6E-02	7.6	15	
H11-H6	3.9E+06	3.2E-02	8.1	22	25
H11-H6	3.0E+06	2.5E-02	8.3	28	
H11-H4_Y104F	1.9E+06	7.2E-02	37.4	10	9
H11-H4_Y104F	2.2E+06	7.4E-02	33.2	9	
H11-H4_Q98R+H100E	5.5E+06	2.4E-02	4.3	29	29
H11-H4_Q98R+H100E	5.2E+06	2.5E-02	4.8	28	
H11-D4*	2.9E+06	2.6E-02	9.0	27	26
H11-D4*	2.9E+06	2.8E-02	9.7	25	
H11-H4*	4.6E+06	2.4E-02	5.5	28	29
H11-H4*	4.5E+06	2.4E-02	5.0	30	

Table S6. Surface area analysis

	PISA(4) Buried Surface Area (Å^2)			Intersurf(5) Buried Surface Area (Å^2)		
	**Nano**	**RBD**	**Total**	**Polar**	**Apolar**	**Total**
H11	614	543	1157	461	718	1179
H11-A10	576	523	1099	423	709	1131
H11-B5	630	590	1220	446	804	1249
H11-H6	595	564	1159	467	724	1191
H11-D4	648	612	1260	568	723	1290
H11-H4	669	607	1276	471	834	1304

References

1. Huo J *et al.* (2020) Neutralizing nanobodies bind SARS-CoV-2 spike RBD and block interaction with ACE2. *Nat Struct Mol Biol* 27(9):846–854.
2. Laskowski RA, Swindells MB (2011) LigPlot+: multiple ligand-protein interaction diagrams for drug discovery. *J Chem Inf Model* 51(10):2778–2786.
3. Huo J *et al.* (2021) A potent SARS-CoV-2 neutralising nanobody shows therapeutic efficacy in the Syrian golden hamster model of COVID-19. *Nat Commun* 12(1):5469.
4. Krissinel E, Henrick K (2007) Inference of macromolecular assemblies from crystalline state. *J Mol Biol* 372(3):774–797.
5. Negi SS, Schein CH, Oezguen N, Power TD, Braun W (2007) InterProSurf: a web server for predicting interacting sites on protein surfaces. *Bioinformatics* 23(24):3397–3399.
